# HIV-induced autophagy suppression in tuberculosis pathogenesis: from mechanisms to therapeutic implications

**DOI:** 10.3389/fimmu.2026.1990644

**Published:** 2026-10-29

**Authors:** Chao Xia, Xiaobo Fan, Fang Cheng, Sen Yang, Xue Zhang, Yemiao Chen

**Affiliations:** 1 Biobank, Clinical Research Center, Chongqing Public Health Medical Center, Chongqing, China; 2 Department of Pharmacy, Chongqing Yubei People’s Hospital, Chongqing, China; 3 Department of Information, Chongqing Public Health Medical Center, Chongqing, China

**Keywords:** ART, autophagy, HIV, host-directed therapy, mycobacterium tuberculosis, viral proteins

## Abstract

Tuberculosis (TB) remains the leading cause of death among people living with HIV (PLWH) despite effective antiretroviral therapy (ART), with HIV infection increasing the risk of active TB by 16 to 27-fold. Even among virologically suppressed individuals on ART, the risk remains 4 to 7-fold higher than in HIV-negative populations, indicating that mechanisms beyond CD4+ T-cell depletion contribute to persistent TB susceptibility. Autophagy is a conserved lysosomal degradation pathway essential for intracellular pathogen clearance and immune regulation, and its dysregulation has emerged as a critical determinant of host-pathogen interactions in HIV/TB co-infection. In this review, we synthesize current evidence suggesting that HIV profoundly suppresses autophagy in key immune cells through the concerted actions of viral proteins, including Nef, Tat, and Env, through the off-target effects of ART drugs. This inhibition may disrupt macrophage antimicrobial functions, including phagosome maturation, xenophagy, and LC3-associated phagocytosis, while also impairing antigen presentation in dendritic cells. These defects may compromise communication between immune cells, attenuate T-cell responses, and create a permissive intracellular niche for Mycobacterium tuberculosis (Mtb) survival and proliferation. We propose an integrated mechanistic framework linking HIV-driven autophagy suppression to enhanced TB susceptibility, highlighting the synergistic interplay between HIV proteins and Mtb evasion strategies. By elucidating these molecular connections, we identify autophagy as a promising therapeutic target and highlight autophagy-directed host-directed therapies as a potential strategy to reduce the persistent TB burden in PLWH and advance toward a cure for both infections.

## 1 Introduction

The co-infection of Human Immunodeficiency Virus (HIV) and Mycobacterium tuberculosis (Mtb) remains a major global public health challenge. In 2025, HIV was responsible for approximately 570, 000 deaths worldwide, with nearly one-third attributed to tuberculosis. TB continues to rank as the leading cause of mortality among people living with HIV (PLWH) (1, 2). The risk of developing active TB disease is 16 to 27 times greater for PLWH than for those without HIV infection (3–5), underscoring the profound intersection of these two epidemics.

Despite decades of research, the molecular and cellular mechanisms through which HIV-1 predisposes individuals to Mtb infection remain incompletely understood (6–8). The classical explanation centers on HIV-1-induced CD4+ T-cell depletion, which compromises host adaptive immunity against Mtb (7, 9). However, this paradigm alone cannot fully account for clinical observations. PLWH exhibit heightened susceptibility to Mtb infection remarkably early after seroconversion, often preceding substantial CD4+ T-cell decline (10, 11). Conversely, even after effective antiretroviral therapy (ART) restores CD4+ and CD8+ T-cell populations and functions, PLWH on ART continue to face a 4- to 7-fold elevated risk of Mtb infection compared to HIV-negative populations (12–14). These discordant observations point toward additional, T-cell-independent mechanisms. In support of this notion, ex vivo studies have demonstrated that HIV infection directly facilitates Mtb replication in macrophages (15), suggesting that HIV can intrinsically enhance Mtb multiplication independent of other immune cell types.

Autophagy is an evolutionarily conserved, lysosome-mediated catabolic pathway that maintains cellular homeostasis by degrading damaged organelles, misfolded proteins, and invading microorganisms (16, 17). Mammalian autophagy comprises three main types: macroautophagy, microautophagy, and chaperone-mediated autophagy (18–20). Macroautophagy is the most extensively characterized form, regulated by autophagy-related genes (ATGs). The process involves activation of the ULK1/2 complex, recruitment of the Beclin 1-containing class III PtdIns3K complex for phagophore nucleation (21), membrane elongation and closure via the ATG12-ATG5-ATG16L1 and LC3 lipidation systems (22), and finally fusion with lysosomes to form autolysosomes where contents are degraded and recycled (23). Beyond bulk degradation, selective forms of autophagy include xenophagy (targeting pathogens), mitophagy (damaged mitochondria), and LC3-associated phagocytosis (LAP) (24, 25). Autophagy is intimately interconnected with innate and adaptive immunity (26), playing critical roles in macrophage phagosome maturation, dendritic cell MHC class II antigen presentation, and CD4+ T cell survival, proliferation, and memory formation (27–30).

In Mtb infection, autophagy has emerged as a central pillar of host innate immune defense (31). Upon Mtb entry into macrophages, bacteria are sequestered in autophagosome-like compartments, initiating autophagic clearance (32, 33). Autophagy promotes maturation of Mtb-containing phagosomes and their fusion with lysosomes, overcoming the pathogen’s blockade of phagolysosomal maturation (34). Additionally, autophagy shapes immune responses by regulating antigen presentation, inflammatory cytokine production, and antimicrobial effector pathways (35–37). Pharmacological (e.g., rapamycin) or physiological (e.g., IFN-γ) activation of autophagy enhances killing of intracellular mycobacteria (38, 39). Conversely, Mtb has evolved strategies to subvert autophagy, including blockade of phagosome maturation, interference with autophagy initiation, and inhibition of autophagosome-lysosome fusion (40).

The relationship between HIV-1 and autophagy is highly dynamic and stage-dependent (41, 42). During early infection, HIV-1 exploits components of the autophagic machinery to facilitate its replication and spread (43, 44). However, as infection progresses to the chronic stage, the virus shifts to actively suppressing autophagy (45). Multiple HIV proteins—including Nef, Tat, and Env—contribute to this suppression through direct interactions with autophagy-related proteins and/or modulation of the mTOR pathway (46). This inhibition prevents sequestration and degradation of viral proteins, facilitating viral persistence and chronic infection (47). Thus, HIV-1 employs a temporally regulated strategy: it exploits autophagy during early infection to enhance replication, and suppresses it during chronic infection to evade host degradation and maintain persistence.

The convergence of HIV-mediated autophagy suppression—particularly in macrophages, CD4+ T cells, and dendritic cells—has profound implications for Mtb control (8, 48). Given that macrophages are the primary host cells for Mtb and autophagy is essential for intracellular killing of this pathogen, HIV-induced autophagic impairment likely creates a permissive environment for Mtb survival and proliferation (49). However, despite growing evidence implicating autophagy in both HIV pathogenesis and anti-TB immunity, a comprehensive synthesis of how HIV-driven autophagy suppression promotes TB pathogenesis is lacking. The intricate relationships among viral proteins, ART agents, autophagic dysfunction, and impaired Mtb control have yet to be consolidated into a unified framework that explains the persistent TB risk in the ART era.

In this review, we address this gap by synthesizing current knowledge across three interrelated domains. First, we delineate the mechanisms by which HIV—through viral proteins and ART drugs—inhibits autophagy in key immune cells and the subsequent immunological disturbances. Second, we provide an overview of the pathways through which autophagy contributes to Mtb containment, including phagosome maturation, xenophagy, LAP, and regulation of inflammatory and apoptotic responses, as well as Mtb evasion strategies. Finally, we integrate these findings into a mechanistic model explaining how HIV-induced autophagy suppression fosters a permissive intracellular environment for Mtb, disrupts adaptive immunity, perpetuates chronic inflammation, and drives the transition from latent infection to active TB disease. Through this integrated perspective, we aim to identify novel therapeutic avenues—particularly autophagy-targeted host-directed therapies—that may mitigate the burden of TB among PLWH and contribute to the goal of achieving a cure for both infections.

## 2 HIV-mediated autophagy inhibition and its immunological consequences

During HIV infection, autophagy degrades viral components and facilitates antigen presentation to elicit antiviral responses (50, 51). However, HIV suppresses autophagic flux in infected cells through viral proteins and ART drugs (45, 52). This impairment promotes viral persistence and immune evasion, and disrupts macrophage, dendritic cell, and T cell functions, contributing to HIV immunopathogenesis (53, 54).

### 2.1 HIV viral proteins that suppress autophagy

HIV has evolved sophisticated strategies to subvert autophagy at multiple steps, primarily through the action of its accessory and regulatory proteins. These proteins target distinct stages of the autophagic process—initiation, elongation, maturation, and lysosomal fusion—thereby ensuring viral persistence and immune evasion (**Figure 1**, **Table 1**).

**Figure 1:**
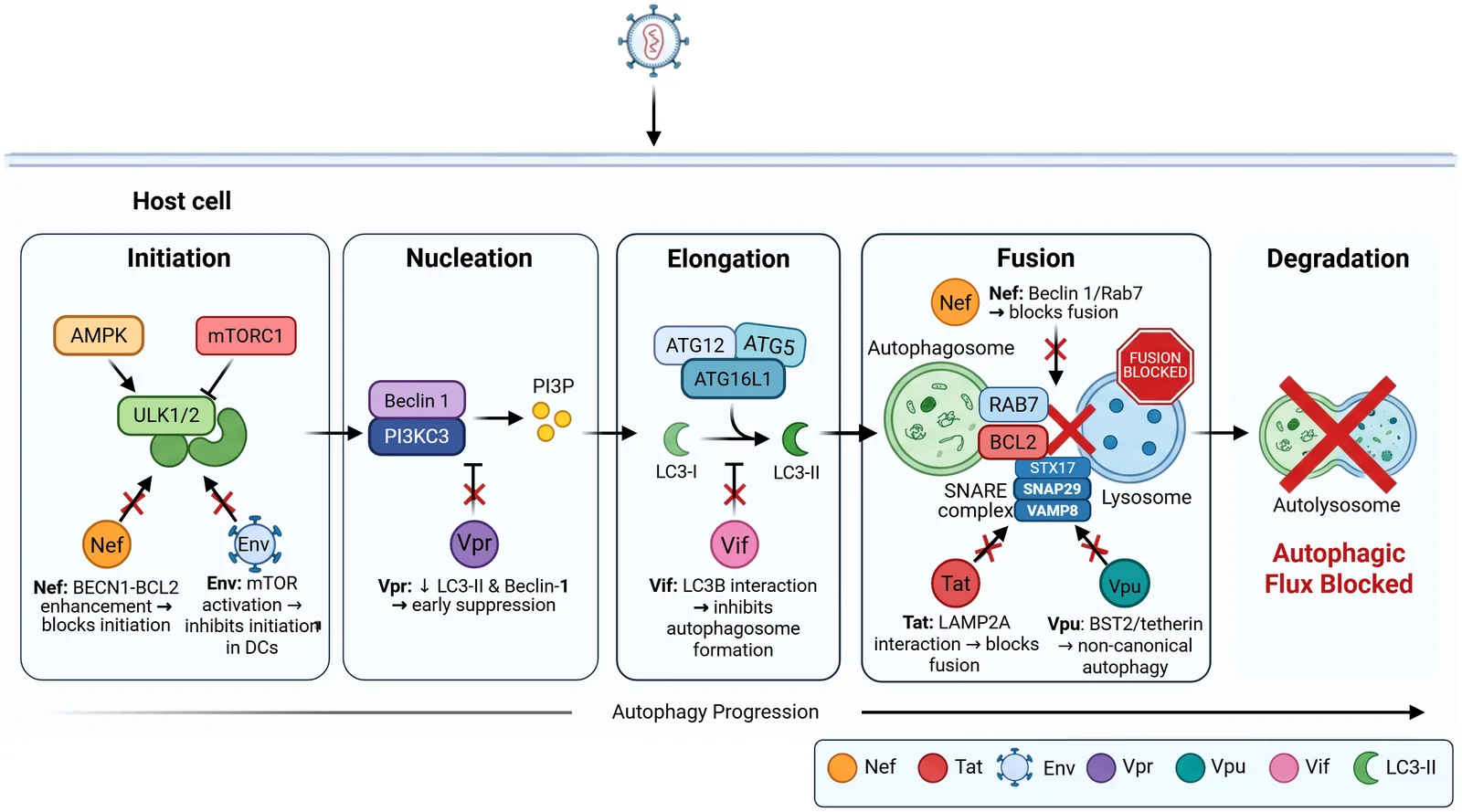
Molecular mechanisms of HIV-mediated autophagy suppression. In the initiation stage, Nef blocks autophagy initiation by enhancing BECN1-BCL2 association via PRKN, while Env activates mTOR to inhibit autophagy in dendritic cells. During nucleation, Vpr rapidly downregulates LC3-II and Beclin-1 levels, suppressing autophagy prior to viral integration. In the elongation stage, Vif interacts with LC3B to inhibit autophagosome formation. The fusion stage is the primary target of multiple HIV proteins: Nef blocks autophagosome-lysosome fusion through interactions with Beclin 1 and Rab7; Tat inhibits fusion via LAMP2A interaction; and Vpu modulates non-canonical autophagy through BST2/tetherin. The cumulative effect of these inhibitory actions across multiple stages results in a complete blockade of autophagic flux, depicted at the degradation stage. (Created by biorender.com).

**Table 1:** HIV viral proteins that suppress autophagy.

HIV protein	Target molecule	Autophagy stage affected	Functional consequence	Functional consequence	References
Nef	Beclin 1, Rab7, BCL2, TFEB	Initiation, Fusion	Autophagosome accumulation, mitochondrial dysfunction	Autophagosome accumulation, mitochondrial dysfunction	(58, 59)
Tat	LAMP2A, LC3-II, p62	Fusion	Autophagic flux blockade, accumulation of misfolded proteins	Autophagic flux blockade	(66, 67)
Env	mTOR	Initiation (in DCs)	Autophagy suppression in dendritic cells, impaired antigen presentation	Autophagy suppression, impaired antigen presentation	(43, 62)
Vpr	LC3-II, Beclin-1	Initiation	Early autophagy suppression	Early autophagy suppression	(69)
Vif	LC3B	Formation	Autophagosome formation inhibition	Autophagosome formation inhibition	(71)
Vpu	BST2/tetherin	Non-canonical autophagy	Modulation of non-canonical autophagy pathway	Modulation of non-canonical autophagy	(72)

#### 2.1.1 Nef

Negative regulatory factor (Nef) is a multifunctional accessory protein that acts as a central mediator of autophagy suppression (55). In primary human fetal astrocytes, adenoviral-mediated Nef expression triggered accumulation of the autophagy markers LC3-II and p62 (SQSTM1), indicating blocked autophagic flux. Mechanistically, Nef disrupted autophagosome-lysosome fusion, thereby helping HIV-1 evade autophagic degradation (56). Subsequent studies revealed that Nef impairs autophagy at the maturation step by interacting with Beclin 1 and Rab7, disrupting TFEB nuclear localization, and compromising lysosomal content (57). Moreover, Castro-Gonzalez et al. demonstrated that Nef blocks autophagy initiation by strengthening the interaction between BECN1 and its inhibitor BCL2, an effect mediated by the E3 ligase PRKN (58). A nef-deficient virus potently induced autophagy, leading to degradation of HIV-1 Gag and inhibition of viral replication, highlighting Nef as the principal autophagy antagonist in HIV infection (59). In Nef-treated primary human astrocytes, autophagosomes underwent accelerated degradation without induction of new autophagosome formation, further underscoring the pleiotropic inhibitory effects of Nef (60). Collectively, Nef functions as a potent autophagy-inhibiting protein that facilitates HIV-1 replication across multiple cell types.

#### 2.1.2 Env

HIV-1 envelope glycoprotein (Env) mediates viral attachment, membrane fusion, and entry. The interaction between Env and autophagy exhibits cell-type-specific duality (61). In CD4+ T cells, prolonged exposure to gp120 induces abnormal autophagic cell death by disrupting calcium homeostasis, leading to excessive autophagy that depletes the immune compartment (62). However, in DCs, HIV-1 Env activates the mTOR pathway, resulting in autophagy inhibition. Blanchet et al. showed that Env-mediated mTOR activation in DCs suppresses autophagy, enhances intracellular HIV-1 accumulation, and facilitates viral transmission to CD4+ T cells. Moreover, this inhibition impairs both innate and adaptive immune responses (43). Early HIV-1 infection suppresses late-stage fusion between late endosomes and autophagosomes by activating mTOR (63). Additionally, HIV-1 Env activates mTORC1 in both productively infected and bystander cells, promoting efficient viral replication and particle production (64). Thus, Env exerts context-dependent effects but predominantly inhibits autophagy in key antigen-presenting cells.

#### 2.1.3 Tat

Trans-activator of transcription (Tat) is essential for viral transcription and replication, but it also plays a critical role in autophagy suppression across multiple cell types (65). In primary cultured rat hippocampal neurons, HIV-1 Tat treatment caused Tat accumulation in endosomes, accompanied by altered endosomal morphology, increased endosomal volume, impaired membrane integrity, elevated pH, reduced enzyme activity, and suppressed autophagy (64). In B103 neuronal cells and mouse primary neurons, Tat suppressed LC3-II and p62 expression in a dose-dependent manner. Mechanistically, Tat colocalized with lysosome-associated membrane protein 2A (LAMP2A) in the cytoplasm, blocking autophagosome-lysosome fusion and leading to accumulation of misfolded proteins and damaged organelles (66). Similar findings were corroborated in cardiomyocytes, where Tat impaired LC3-II and p62 expression, abrogating autophagosome formation and maturation (67). Importantly, Tat inhibits autophagy in CD4+ T lymphocytes by interacting with p62/SQSTM1 in a ubiquitin-independent manner (68). Thus, Tat exhibits broad suppressive effects on autophagy across neurons, cardiomyocytes, and immune cells, contributing to HIV-associated neurocognitive disorders (HAND), cardiac disease, and immune dysfunction.

#### 2.1.4 Other viral proteins

Additional HIV proteins contribute to autophagy inhibition. Viral Protein R (Vpr) rapidly downregulates LC3-II and Beclin-1 levels in CD4+ T cells during early infection, suppressing autophagy prior to viral integration (69). Vpr also reduces lysosomal acidification, disrupts lysosomal localization, and modulates proteins involved in lysosomal maturation, leading to SNCA protein accumulation in neurons and neurodegenerative pathology (70). Viral Infectivity Factor (Vif) directly interacts with LC3B, binding both precursor LC3 and the cleaved form, with the glycine 120 site on phosphatidylethanolamine-bound autophagosomes being critical. Vif suppresses autophagy during HIV-1 infection (71). Vpu contributes to non-canonical autophagy by modulating BST2/tetherin restriction (72). Furthermore, the autophagy-related protein ATG5 has been identified as a central mediator of a non-canonical autophagy pathway hijacked by HIV Vpu to weaken the host response, involving the recognition and binding of virus-tethered BST2 (73). Together, these viral proteins ensure comprehensive blockade of the autophagic pathway.

### 2.2 ART drugs that inhibit autophagy

While ART is life-saving, many antiretroviral drugs exert off-target effects on autophagy, contributing to long-term toxicities and incomplete immune recovery. This effect is drug-class specific.

#### 2.2.1 Nucleoside reverse transcriptase inhibitors

Zidovudine (AZT/ZDV) blocks autophagosome-lysosome fusion in myocytes and T cells, inhibiting late-stage autophagy. This leads to accumulation of dysfunctional mitochondria, elevated ROS production, and cellular toxicity (74). In a rat model, AZT induced mitochondrial DNA depletion in the liver while concurrently suppressing autophagy—evidenced by elevated AKT/mTOR activity, reduced AMPK/ULK1 activity, decreased lysosomal numbers (LAMP2), reduced TFEB levels, and increased autophagosome abundance (LC3-II) and p62 accumulation (75). Stavudine (d4T) inhibits autophagy in adipocytes and fibroblasts, driving lipodystrophy and insulin resistance (76). Tenofovir disoproxil fumarate (TDF) combined with emtricitabine (FTC) and integrase inhibitors disrupts lysosomal acidification and impairs autophagosome maturation in microglia, compromising clearance of aggregated proteins and contributing to HAND (77). Recent studies indicate that different antiretroviral drugs impact autophagy differentially in primary human astrocytes, representing alterations of cellular homeostasis (78).

#### 2.2.2 Protease inhibitors

Lopinavir (LPV), ritonavir (RTV), and saquinavir attenuate autophagy in U87 cells via the p38 MAPK pathway, with increased p62 and decreased LC3II/LC3I ratios (79). Lopinavir/ritonavir activate endoplasmic reticulum stress and inhibit autophagic activity, inducing dysregulation of lipid metabolism in adipocytes (80).

#### 2.2.3 Integrase inhibitors

Dolutegravir (DTG)-based regimens impair lysosomal function in microglia, reduce autophagic flux, and increase ROS (77). Similarly, raltegravir (RAL)-based regimens decrease LC3-II and Beclin-1 expression in astrocytes, leading to neuroinflammation (60).

### 2.3 Immunological consequences of autophagy inhibition

The dual inhibition of autophagy by HIV proteins and ART drugs produces profound immunological derangements across multiple immune cell lineages, collectively undermining both innate and adaptive immunity. In macrophages, HIV-induced autophagy impairment—mediated primarily by Nef’s interaction with Beclin 1—compromises the cell’s ability to clear intracellular pathogens and dysfunctional mitochondria, leading to accumulation of reactive oxygen species and inflammatory mediators (53, 81). In CD4+ T cells, autophagy inhibition disrupts mitochondrial quality control and promotes apoptotic cell death, exacerbating the progressive depletion of this critical cell population that characterizes HIV infection (69, 82). Notably, elite controllers and long-term nonprogressors exhibit higher autophagic activity, suggesting that preserved autophagy may contribute to better clinical outcomes (45). In dendritic cells, HIV Env-mediated mTOR activation suppresses autophagy and reduces MHC class II antigen presentation, thereby limiting the priming of HIV-specific CD4+ T cell responses (43). ART regimens further compound these defects by exerting additional inhibitory effects on autophagic flux in various cell types (83). Collectively, the convergence of HIV- and ART-driven autophagy suppression cripples the functional integrity of macrophages, T cells, and dendritic cells, contributing to persistent immune dysfunction, chronic inflammation, and the impaired immunological recovery observed in a subset of ART-treated individuals.

Having established how HIV suppresses autophagy in key immune cells, we next examine the essential roles of autophagy in controlling Mtb infection, and how these defenses are subverted by the pathogen.

Together, these viral proteins and ART drugs target multiple stages of autophagy—initiation, elongation, maturation, and lysosomal fusion—in macrophages, dendritic cells, CD4+ T cells, and other cell types, indicating that HIV-mediated autophagy suppression is broad and multi-layered. Notably, ART drugs such as NRTIs, PIs, and INIs also inhibit autophagy at distinct steps, further broadening the overall suppression.

## 3 The role of autophagy in controlling mycobacterium tuberculosis infection

Autophagy plays a critical role in host defense against Mtb, the etiological agent of TB. This section provides a comprehensive overview of the molecular mechanisms by which autophagy contributes to anti-TB immunity and the countermeasures employed by Mtb to evade this host defense system, establishing the foundation for understanding how HIV-induced autophagy suppression exacerbates TB susceptibility.

### 3.1 Autophagy as a defense mechanism against Mtb

Upon inhalation, Mtb reaches the alveolar space and is phagocytosed by macrophages, which represent the first line of host defense, and following phagocytosis, Mtb resides within phagosomes where it would normally be eliminated through phagolysosomal fusion (84–86). However, Mtb has evolved mechanisms to arrest phagosome maturation, preventing acidification and lysosomal delivery and thereby establishing a permissive intracellular niche, yet autophagy provides a critical countermeasure by promoting the maturation of Mtb-containing phagosomes and facilitating their fusion with lysosomes to overcome the pathogen’s maturation blockade (87). This section details the multiple pathways through which autophagy contributes to the control of Mtb infection (**Figure 2**).

**Figure 2:**
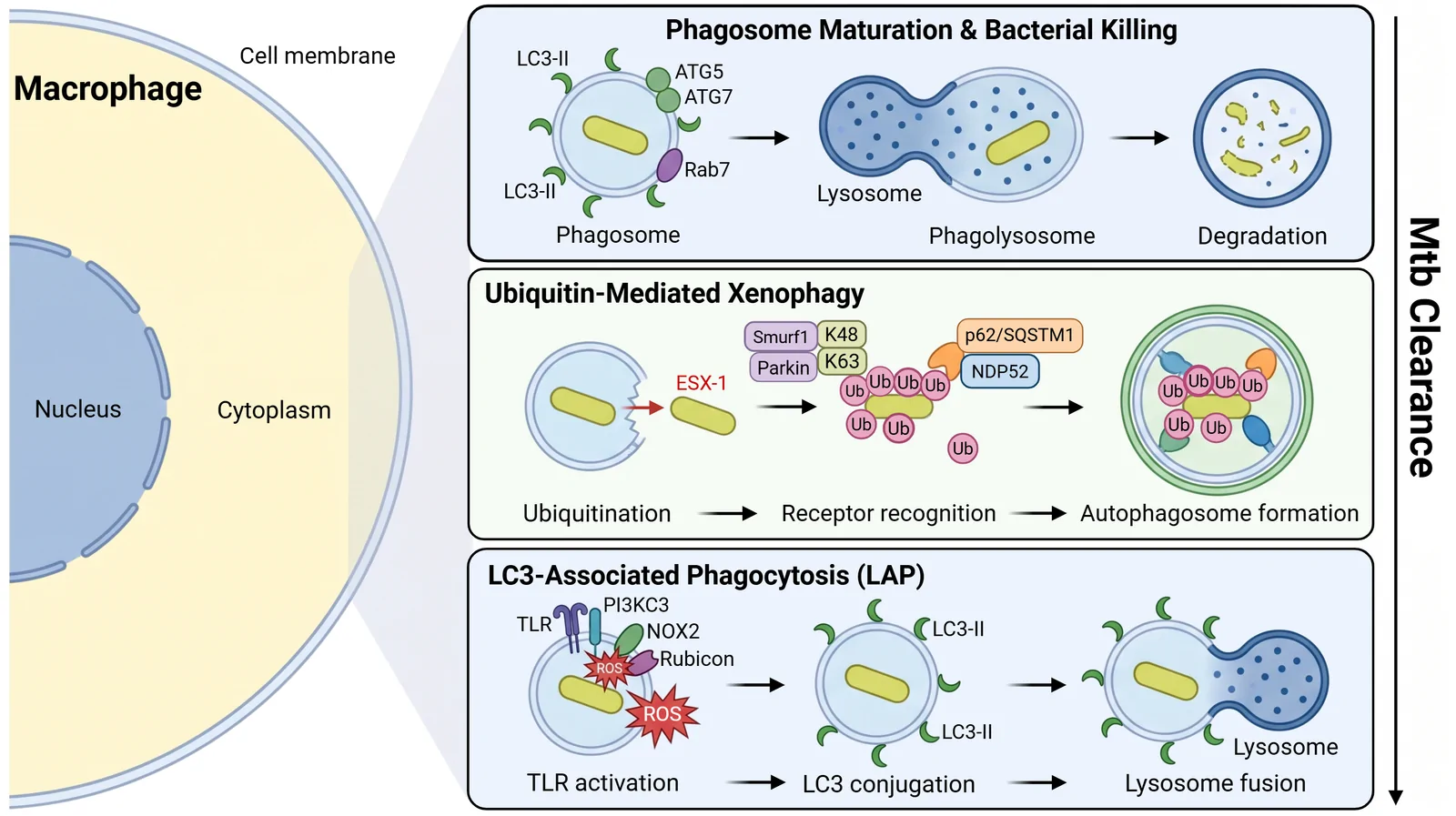
Autophagy defense pathways against *Mycobacterium tuberculosis* (Mtb) infection in macrophages. In Pathway A (phagosome maturation and bacterial killing), Mtb-containing phagosomes recruit LC3-II and ATG proteins, which accelerate Rab7-mediated lysosomal fusion to form phagolysosomes where acidification and hydrolases mediate bacterial degradation. In Pathway B (ubiquitin-mediated xenophagy), Mtb utilizes the ESX-1 secretion system to escape into the cytosol, where it is tagged with ubiquitin chains by E3 ligases Smurf1 (K48-linked) and Parkin (K63-linked). Autophagy receptors p62/SQSTM1 and NDP52 recognize ubiquitinated Mtb and bridge it to LC3-II on developing autophagosomes, leading to lysosomal degradation. In Pathway C (LAP), TLR activation recruits the PI3KC3 complex and Rubicon to single-membrane phagosomes, generating PI3P and ROS via NOX2, which drives LC3 lipidation and promotes rapid phagosome-lysosome fusion independently of autophagosome formation. Together, these pathways constitute the core autophagic defense against Mtb. (Created by biorender.com).

#### 3.1.1 Autophagy in phagosome maturation and bacterial killing

Following Mtb infection, activation of pattern recognition receptors such as Dectin-1 and Toll-like receptors triggers the recruitment of LC3-II to the Mtb-containing phagosomal membrane. This process requires ATG5, ATG7, and other ATG proteins. These proteins accelerate the recruitment of maturation markers such as Rab7 and promote fusion with lysosomes, thereby enhancing bactericidal activity (84, 88, 89). Autophagy also enhances the production of reactive oxygen species and reactive nitrogen species, contributing to multi-level killing, and furthermore regulates cytokine secretion and antigen processing to modulate both innate and adaptive immunity (90). Overall, autophagy synergizes with phagosomes to encapsulate pathogens, promote maturation, and facilitate bactericidal degradation, although Mtb has evolved strategies to block phagosome maturation and fusion, enabling cytoplasmic escape and inhibiting autophagy (91). Multiple lines of evidence support the protective role of autophagy in anti-TB immunity: activation of autophagy via pharmacological agents such as rapamycin or physiological stimuli such as IFN-γ enhances the killing of intracellular Mtb in macrophages (38), whereas genetic ablation of core autophagy genes, such as ATG5, ATG16L1, or ATG7, in myeloid cells increases host susceptibility to M. tuberculosis and promotes bacterial growth (92).

#### 3.1.2 Ubiquitin-mediated xenophagy

Xenophagy is a selective form of autophagy that targets intracellular pathogens. During Mtb infection, the bacterium utilizes its ESX-1 type VII secretion system to permeabilize the phagosomal membrane, allowing cytosolic sensors to detect bacterial components within minutes of infection. Within hours, a substantial fraction of intracellular Mtb bacilli are surrounded by ubiquitin, LC3, and selective autophagy adaptors such as p62 and NDP52, which target the bacteria to autophagosomes for degradation. In the absence of this selective autophagy targeting, Mtb survives and replicates to a significantly higher degree (93).

Two E3 ubiquitin ligases play critical roles: Smurf1 primarily mediates K48-linked ubiquitination while Parkin mainly participates in K63-linked ubiquitination, and together they coordinate the tagging of intracellular pathogens and promote selective autophagy (94). Smurf1 is essential for anti-Mtb immunity and also regulates pulmonary inflammation, making it an indispensable regulator of host defense (95), and additionally, ubiquitin can bind directly to the Rv1468c protein on the surface of Mtb, triggering xenophagy independently of other ubiquitination pathways (96), while the cytosolic DNA sensor cGAS can also identify Mtb DNA in the cytoplasm to trigger autophagy and type I interferon responses that further restrict bacterial growth (97).

#### 3.1.3 LAP

LAP is a non-canonical autophagy pathway that involves the conjugation of LC3 to single-membrane phagosomes, originating from plasma membrane invaginations and being distinct from canonical autophagy (98). Mtb can activate Toll-like receptors, which recruit the PI3KC3 (VPS34) complex comprising VPS34, Beclin-1, UVRAG, and Rubicon to single-membrane phagosomes to generate PI3P, while Rubicon stabilizes the NOX2 complex to drive ROS production. After which various ATG proteins are recruited to the phagosomal membrane to catalyze LC3-II formation and its surface modification, ultimately allowing the phagosome to fuse with the lysosome to clear the pathogen (25). The LAP pathway effectively bypasses Mtb-mediated inhibition of lysosomal maturation, providing a critical backup mechanism for bacterial elimination (99). Studies have demonstrated that macrophages with LAP pathway abnormalities are less effective at controlling bacterial infections such as Legionella pneumophila, Staphylococcus aureus, and Mtb, underscoring the importance of this pathway in anti-TB host defense (100). Another key role of LAP is to aid antigen presentation via MHC class II molecules for adaptive immunological response activation, and it has been observed that the absence of LAP impairs the presentation of pathogen antigens on MHC class II molecules, thereby impairing anti-infection immunity (101).

### 3.2 Mtb evasion strategies against autophagy

Despite the potent anti-mycobacterial functions of autophagy, Mtb has evolved a diverse array of mechanisms to evade autophagic clearance, ensuring its intracellular survival and persistent infection. These evasion strategies operate at multiple levels, targeting various stages of the autophagic pathway (102) (**Table 2**).

**Table 2:** Mtb Evasion strategies against autophagy.

Mtb factor	Target molecule/pathway	Autophagy stage affected	Functional consequence	References
LAM	PI3P-dependent trafficking	Phagosome maturation	Blocks phagosome maturation	(87, 104)
SapM	PI3P (hydrolysis)	Phagosome maturation	Prevents PI3P accumulation	(105, 106)
PknG	Phagosome acidification	Maturation	Interferes with acidification and maturation	(107)
Coronin 1	Lysosomal delivery	Fusion	Specifically blocks lysosomal delivery	(108)
Eis	JNK-ROS signaling	Initiation	Suppresses autophagy initiation	(110)
LprE	Autophagy/histone expression	Initiation	Inhibits autophagy expression	(111)
PE_PGRS47/20	Rab1A	Initiation	Blocks autophagy initiation	(113)
PPE51	TLR2-ERK1/2	Initiation	Suppresses autophagy	(114)
CpsA	NOX2/ROS	LAP	Protects Mtb from LAP-mediated killing	(119)

Mtb arrests phagosome maturation by residing within modified phagosomes that fail to acquire Rab5 and Rab7, maintaining a neutral pH that prevents acid-dependent hydrolase activation (103). This arrest is mediated by multiple mycobacterial factors: (a) the cell wall glycolipid lipoarabinomannan (LAM) interferes with PI3P-dependent trafficking (87, 104); (b) the acid phosphatase SapM hydrolyzes PI3P on the phagosome membrane (105, 106); (c) the protein kinase PknG interferes with phagosome acidification and maturation, and Coronin 1 specifically blocks lysosomal delivery (107, 108). Macrophages lacking LRG-47 have reduced acidification capacity, further highlighting the importance of phagosome maturation in anti-TB immunity (109).

Mtb also suppresses autophagy at the initiation stage: the Eis protein inhibits macrophage autophagy by suppressing JNK-ROS signaling (110); the LprE lipoprotein inhibits autophagy and histone expression (111); and the PE/PPE and PE_PGRS protein families, which are unique to the Mtb genome and account for approximately 10% of its coding capacity, modulate host autophagy (112). Furthermore, PE_PGRS47 and PE_PGRS20 interact with the host small GTPase Rab1A to block autophagy initiation (113), while PPE51 inhibits autophagy by suppressing TLR2-ERK1/2 signaling (114), and PE_PGRS41 indirectly suppresses autophagy-related defense responses (115).

In addition to inhibiting initiation, Mtb blocks autophagosome-lysosome fusion. The ESX-1 secretion system mediates phagosomal damage and cytosolic release of bacterial components, while the SecA2 pathway exports effectors that arrest phagosome and autophagosome maturation (116, 117). Mtb can also interfere with Rab7 recruitment to the phagosome, thereby evading phagosome-lysosome fusion (118). Furthermore, Mtb counteracts xenophagy and LAP: the CpsA protein protects Mtb from NOX2-dependent ROS and LAP-mediated killing (119), and the bacterium may secrete factors that inhibit the ubiquitination of bacterial proteins or the recruitment of autophagy adaptor proteins (96).

In summary, autophagy serves as a critical host defense mechanism against Mtb through multiple interconnected pathways, including phagosome maturation, xenophagy, LC3-associated phagocytosis, and regulation of inflammation and apoptosis, yet Mtb has evolved a sophisticated repertoire of countermeasures that target virtually every stage of the autophagic pathway, enabling its intracellular persistence. The therapeutic targeting of autophagy holds promise for TB treatment.

These findings demonstrate that HIV and Mtb act synergistically to suppress autophagy beyond the level achieved by either pathogen alone. This synergistic suppression creates a permissive intracellular environment for Mtb survival and replication, and highlights the potential of autophagy restoration as a therapeutic strategy to reduce TB risk in PLWH.

## 4 How HIV-induced autophagy inhibition promotes tuberculosis pathogenesis: a mechanistic synthesis

Building on the preceding sections, we now propose a cohesive mechanistic framework linking HIV-mediated autophagy suppression to enhanced susceptibility and progression of tuberculosis. This synthesis integrates the specific molecular nodes of autophagy targeted by HIV, the essential autophagic processes required for Mtb control, and the clinical and experimental evidence supporting this causal cascade. The core argument is that HIV, through both viral proteins and ART drugs, profoundly impairs autophagic flux in macrophages and other immune cells, and this impairment cripples the very mechanisms that are essential for containing and eliminating Mtb, thereby creating a permissive environment for bacterial persistence, proliferation, and dissemination (**Figure 3**).

**Figure 3:**
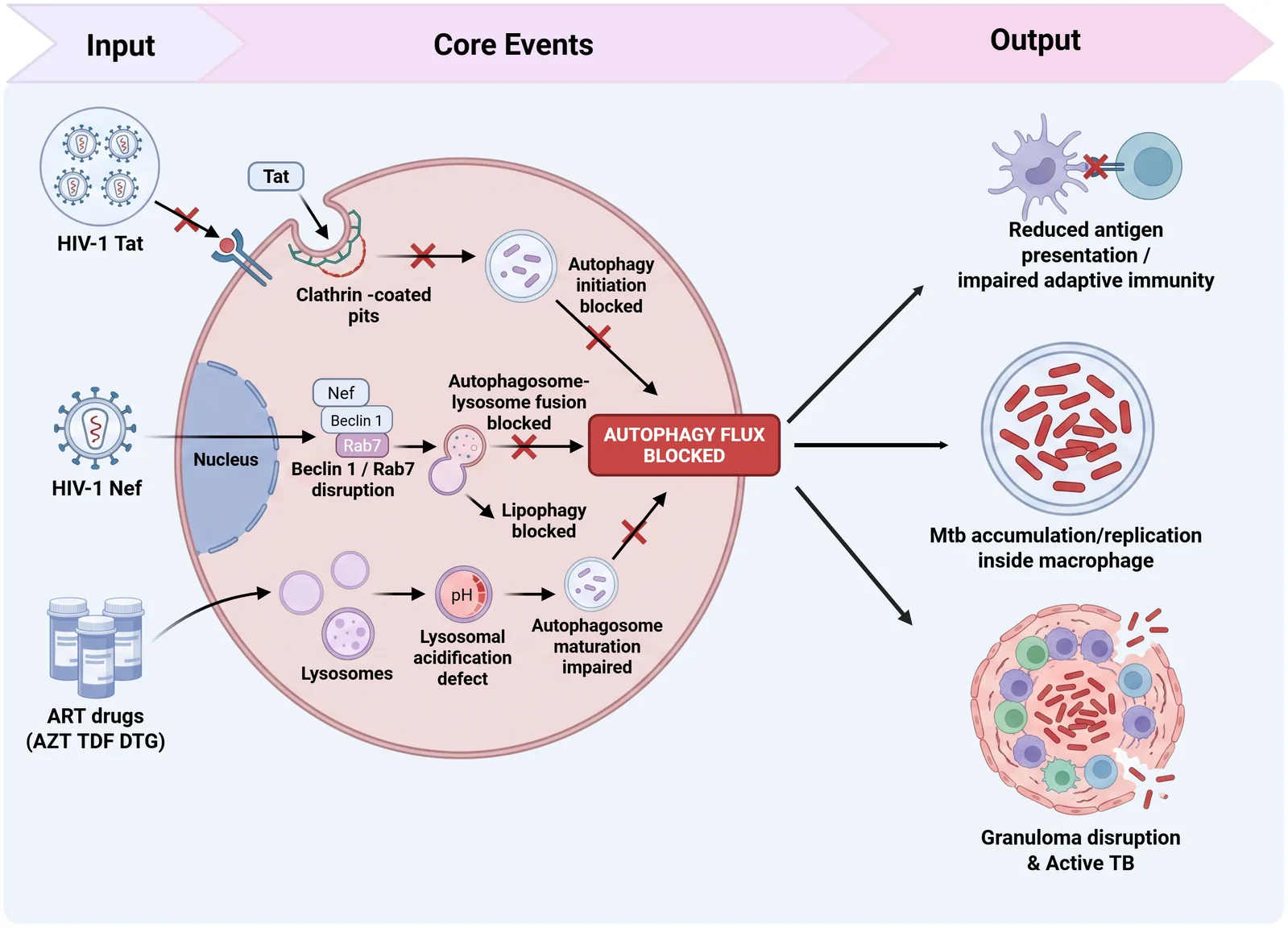
Integrated model of HIV-induced autophagy suppression and its contribution to Mtb pathogenesis. This schematic illustrates the mechanistic framework by which HIV-1 viral proteins (Tat, Nef, Env) and antiretroviral (ART) drugs (AZT, TDF, DTG) converge to disrupt autophagic flux in macrophages, thereby promoting TB progression. Tat binds to plasma membrane PI(4, 5)P2 and undergoes palmitoylation, inhibiting AP-2 adaptor complex recruitment and blocking clathrin-mediated endocytosis, which prevents autophagy initiation; Nef interacts with Beclin 1 and Rab7 to block autophagosome-lysosome fusion and inhibits lysosomal function through TFEB phosphorylation, while also blocking lipophagy to allow lipid droplet accumulation that provides a lipid source for Mtb; and ART drugs induce lysosomal acidification defects that impair autophagosome maturation. These three pathways converge to cause a complete blockade of autophagic flux, resulting in reduced antigen presentation and impaired adaptive immunity, extensive accumulation and replication of Mtb within macrophages, and ultimately granuloma disruption leading to active TB disease. This integrated model explains how HIV-induced autophagy suppression creates a permissive intracellular environment for Mtb survival, proliferation, and dissemination, driving the transition from latent infection to active TB. (Created by biorender.com).

### 4.1 HIV viral proteins suppress autophagy and promote TB pathogenesis

HIV proteins directly suppress autophagy in macrophages, creating a permissive intracellular environment for Mtb survival and proliferation. Among these proteins, Tat has emerged as a key factor promoting Mtb multiplication. Beyond its canonical role in transcription, Tat is secreted by infected cells, binds to phosphatidylinositol-4, 5-bisphosphate at the plasma membrane, and undergoes palmitoylation, enabling it to inhibit recruitment of the AP-2 adaptor complex and block clathrin-mediated endocytosis. This, in turn, suppresses autophagy initiation and prevents the degradation of intracellular pathogens such as Mtb. Tat-mediated autophagy inhibition also blocks lipophagy, facilitating Mtb access to lipids that fuel its proliferation. In zebrafish larval models, exposure to Tat significantly increased susceptibility to mycobacterial infection and enhanced granuloma formation (120). Nef also plays a central role in HIV/TB coinfection. By blocking autophagosome–lysosome fusion and impairing lysosomal function through TFEB phosphorylation, Nef compromises macrophage-mediated clearance of Mtb and promotes bacterial survival (57, 58). Nef-mediated autophagy suppression may therefore directly impair the capacity of macrophages to control Mtb, contributing to the increased susceptibility to TB observed in people living with HIV (PLWH). Env, in turn, activates the mTOR pathway to suppress autophagy in dendritic cells, thereby impairing antigen presentation and innate immune responses (43). Importantly, this suppression is not transient but persists during chronic infection and even under ART, as Tat continues to be secreted and Nef remains expressed in infected cells. Collectively, these findings demonstrate that multiple HIV-1 proteins converge on the autophagy pathway at the initiation, maturation, and lysosomal stages, suppressing autophagy and promoting Mtb pathogenesis.

### 4.2 ART-induced autophagy inhibition further compromises Mtb control

While ART effectively suppresses HIV replication, many antiretroviral drugs exert off-target inhibitory effects on autophagy, adding a second layer of impairment independent of viral proteins (60, 74). Nucleoside reverse transcriptase inhibitors such as AZT block autophagosome-lysosome fusion in myocytes and T cells, inhibiting late-stage autophagy and leading to the accumulation of dysfunctional mitochondria and reactive oxygen species (121). The combination of TDF, FTC, and DTG impairs lysosomal function and autophagosome maturation in microglia, while TDF/FTC/raltegravir (RAL) inhibits autophagy in human astrocytes by blocking autophagosome formation (77, 83). This drug-induced suppression adds a second layer of autophagy impairment that operates independently of viral proteins. Consequently, PLWH on ART may still exhibit defective macrophage autophagy due to the medications themselves, which may explain why even patients with good viral suppression and CD4+ recovery have higher TB incidence than HIV-negative controls.

### 4.3 Impaired antigen presentation

The impairment of autophagy in antigen-presenting cells has profound consequences for adaptive immunity. Autophagy is essential for efficient MHC class II antigen presentation in macrophages and dendritic cells (122). HIV-mediated autophagy inhibition, especially via Env-induced mTOR activation in dendritic cells, reduces the processing and presentation of Mtb antigens, thereby blunting Mtb-specific CD4+ T cell responses (123). This impairs granuloma maintenance and effector function, allowing Mtb to disseminate (124). Additionally, autophagy inhibition in CD4+ T cells promotes T cell apoptosis and mitochondrial dysfunction, further depleting the Mtb-specific T cell pool (125). Thus, HIV-induced autophagy suppression simultaneously cripples adaptive immunity through defective antigen presentation, collectively creating an immunological environment that favors Mtb dissemination and disease progression.

### 4.4 Synergy between HIV and Mtb in suppressing autophagy

Co-infection creates a vicious cycle in which HIV inhibits autophagy, allowing Mtb to replicate and secrete its own effectors that further suppress autophagy, such as the PE/PPE and PE_PGRS proteins (126, 127). Thus the combined effect of HIV and Mtb proteins may result in a deeper and more sustained autophagy block than either pathogen alone, accelerating disease progression. Experimental co-infection model demonstrate that Mtb exacerbates HIV-induced autophagy suppression and vice versa (128). Furthermore, vitamin D supplementation, which induces autophagy, has been associated with reduced TB risk in some studies (129). Taken together, these observations indicate that HIV and Mtb act synergistically to suppress autophagy beyond the level achieved by either pathogen alone, and that therapeutic strategies aimed at restoring autophagic flux may help break this vicious cycle and reduce TB risk in people living with HIV.

In summary, HIV infection of macrophages and T cells suppresses autophagy through Nef, Tat, Env, Vpr, and ART drugs. This suppression cripples phagosome maturation, xenophagy, LC3-associated phagocytosis, and antigen presentation. Consequently, Mtb survives and replicates intracellularly, acquires lipids from accumulated lipid droplets, and disseminates due to impaired adaptive immunity. ART, while controlling viremia, may not reverse autophagy deficits due to persistent Tat and drug side effects, thereby maintaining TB risk.

## 5 Conclusions

Autophagy inhibition has emerged as a central feature of HIV infection, driven by both viral proteins (Nef, Tat, Env, Vpr, Vif, Vpu) and ART drugs (NRTIs, PIs, INIs). This review has systematically examined the evidence that this inhibition may compromise the host’s ability to control Mtb by blocking phagosome maturation, xenophagy, LC3-associated phagocytosis, and antigen presentation, while simultaneously promoting inflammation and creating a nutrient-rich environment for bacterial proliferation. The discovery of Tat-mediated blockade of clathrin-mediated endocytosis suggests a compelling molecular link between HIV and TB susceptibility, potentially explaining the persistent risk even in ART-treated individuals. The integrated model we propose—wherein HIV-induced autophagy suppression may cripple macrophage bactericidal functions, impair adaptive immunity, sustain chronic inflammation, and synergize with Mtb-encoded evasion mechanisms—offers a comprehensive framework for understanding the disproportionately high burden of tuberculosis in people living with HIV.

In conclusion, by integrating our understanding of HIV-induced autophagy suppression with the essential role of autophagy in anti-TB immunity, we may help develop novel strategies to reduce the devastating burden of tuberculosis in people living with HIV. The mechanistic framework presented in this review not only helps explain the epidemiological observations of persistent TB risk in the ART era but also identifies actionable therapeutic targets. Continued interdisciplinary research at the intersection of virology, immunology, and autophagy biology will be essential for translating these insights into clinical practice, ultimately moving toward improved outcomes and, potentially, a cure for both infections.

Although the evidence supporting HIV-induced autophagy suppression as a driver of TB pathogenesis is compelling, several questions remain unresolved. The relative contributions of individual viral proteins and ART drugs *in vivo* require further investigation. Moreover, the extent to which restoring autophagy can reverse established immune dysfunction in individuals receiving ART remains unclear. These knowledge gaps underscore the need for further research.
